# Distribution Optimization: An evolutionary algorithm to separate Gaussian mixtures

**DOI:** 10.1038/s41598-020-57432-w

**Published:** 2020-01-20

**Authors:** Florian Lerch, Alfred Ultsch, Jörn Lötsch

**Affiliations:** 1 DataBionics Research Group, University of Marburg, Hans - Meerwein - Straße 22, 35032 Marburg, Germany; 2 Institute of Clinical Pharmacology, Goethe-University, Theodor - Stern - Kai 7, 60590 Frankfurt am Main, Germany; 3 Fraunhofer Institute of Molecular Biology and Applied Ecology - Project Group Translational Medicine and Pharmacology (IME-TMP), Theodor - Stern - Kai 7, 60590 Frankfurt am Main, Germany

**Keywords:** Data processing, Functional clustering

## Abstract

Finding subgroups in biomedical data is a key task in biomedical research and precision medicine. Already one-dimensional data, such as many different readouts from cell experiments, preclinical or human laboratory experiments or clinical signs, often reveal a more complex distribution than a single mode. Gaussian mixtures play an important role in the multimodal distribution of one-dimensional data. However, although fitting of Gaussian mixture models (GMM) is often aimed at obtaining the separate modes composing the mixture, current technical implementations, often using the Expectation Maximization (EM) algorithm, are not optimized for this task. This occasionally results in poorly separated modes that are unsuitable for determining a distinguishable group structure in the data. Here, we introduce “Distribution Optimization” an evolutionary algorithm to GMM fitting that uses an adjustable error function that is based on chi-square statistics and the probability density. The algorithm can be directly targeted at the separation of the modes of the mixture by employing additional criterion for the degree by which single modes overlap. The obtained GMM fits were comparable with those obtained with classical EM based fits, except for data sets where the EM algorithm produced unsatisfactory results with overlapping Gaussian modes. There, the proposed algorithm successfully separated the modes, providing a basis for meaningful group separation while fitting the data satisfactorily. Through its optimization toward mode separation, the evolutionary algorithm proofed particularly suitable basis for group separation in multimodally distributed data, outperforming alternative EM based methods.

## Introduction

Finding subgroups in biomedical data is a key task in biomedical research and precision medicine. In complex multifactorial data, this is achieved by various methods such as cluster analysis, principal component analysis or several implementations of unsupervised machine learning. However, simpler one-dimensional data, such as many different readouts from cell experiments, preclinical or human laboratory experiments or clinical signs also often reveal a distribution that is more complex than a single mode. Indeed, various tests for multimodal distributions of one-dimensional data have been proposed (for a review, see^1^), emphasizing the relevance of the problem.

Given the frequency of normally distributed biomedical data, Gaussian mixtures play a particularly important role in the multimodal distribution of one-dimensional data that are composed of different subgroups generated by the influence of various biological factors on the research readout. A Gaussian mixture model (GMM), expressed as $p(x|\Theta )$, is defined as the weighted sum of M >1 components $p(x|{\theta }_{m})$ in ${\mathbb{R}}$,

1 $$p(x|\Theta )={\sum }_{m=1}^{M}{\alpha }_{m}p(x|{\theta }_{m}),$$

where $x\in X=\{{x}_{1},\ldots ,{x}_{N}\}$ is a data sample and $\Theta =({\alpha }_{1},\ldots ,{\alpha }_{M};{\theta }_{1},\ldots ,{\theta }_{M})$ the parameterization of the GMM with *a*_*m*_ corresponding to the weight of each component $m=1,\ldots ,M$. Each component $p(x|{\theta }_{m})$ is represented as a normal distribution with parameters ${\theta }_{m}=({\mu }_{m},{\sigma }_{m})$, i.e., mean value und the standard deviation.

However, although GMM fitting is often aimed at obtaining the separate modes composing the mixture, current technical implementations are not optimized for this task. The common approach to model GMM is maximizing the likelihood for a given dataset, using some variant of Expectation Maximization (EM)^2,3^ or Markov-chain Monte-Carlo algorithm^4^. However, one problem with maximizing only the model’s likelihood is that there may be multiple models with high likelihood that pass statistical tests but are structurally different and may be more suited for modelling classes. A possible solution has been proposed as restricted EM^5^, which can lead to different solutions, but tends to be complicated and restrains are limited to relations within the parameters of a GMM, instead of general properties of the GMM as whole. Thus, maximizing the likelihood, or an equivalent measure, of the GMM is a common approach to GMM fitting that has several limitations that have been incompletely addressed. Moreover, it does not directly address the separation of the modes of which the GMM is composed.

Therefore, we propose an evolutionary algorithm for GMM fitting that uses an adjustable error function that is based on χ^2^ statistics and the probability density function. The algorithm can be directly targeted at the separation of the modes of the mixture by employing additional criterion for the degree by which single modes overlap. Using Bayesian decision borders^6^, a simple and effective classifier can be created that allows a valid association of data set members to distinct groups defined by the mode of the Gaussian mixture.

## Methods

### Distribution optimization algorithm

**Figure 1:**
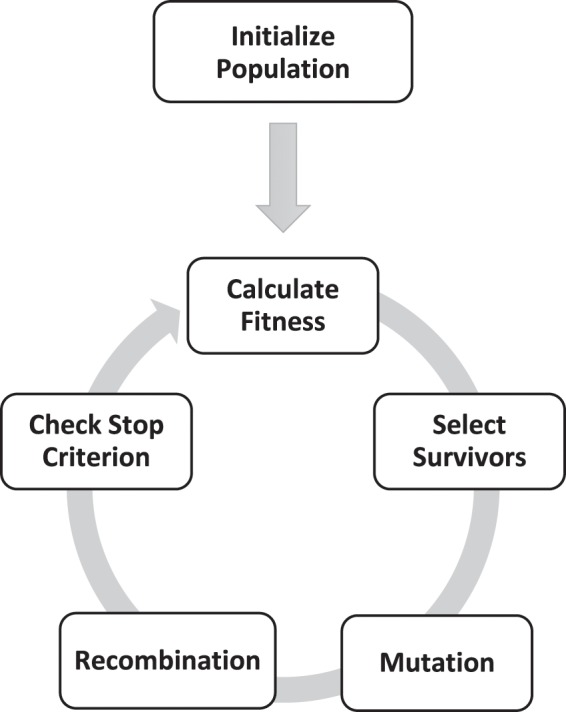
Workflow diagram of the “Distribution Optimization” evolutionary algorithm.

“Distribution Optimization” minimizes a given distribution error by utilizing an evolutionary algorithm. Hence, a “population” of GMMs is processed through many iterations with multiple phases, which mutate, filter (select) and recombine the GMMs (Fig. 1). In general, after randomly initializing a first “generation” of GMM components, in iteration GMM parameters are randomly changed and recombined and individuals with better fitness are chosen, to finally form a new “generation” of GMMs.

The first phase, the initialization step, creates the population of GMMs and draws a value for every parameter of every GMM. It therefore depends on the minima and maxima of every structural model parameter. The individuals are drawn from a uniform distribution within those limits. The mean and standard deviations are drawn within the minimum and maximum of the original data. The weights of each component are randomly drawn from [0,1] and divided by their sum so that they add up to a value of 1.

The core of the “Distribution Optimization” algorithm is its error function, which in its negated form also serves as the fitness function. It is defined as the χ^2^ value, which can be used to compare a theoretical and an empiric distribution. The data space is split into B adjacent intervals (B being derived from standard deviation^7,8^ for our datasets) with ${k}_{j}$ being the fraction of empirical data points falling into the j^th^ interval and *P*_*j*_ being the density of the GMM on the same interval. The error is then defined as:

2 $${{\rm{\chi }}}^{2}={\sum }_{j=1}^{B}\frac{{k}_{j}-{p}_{j}N}{{p}_{j}N}$$

In addition to the χ^2^ value, a second error term is included as the so-called “overlap error”. It approximates the fraction of the area overlapped between modes, relative to the total area under the total density curve. The overall overlap error of a GMM is the highest overlap error among all components, given as

3 $$OverlapErro{r}_{m}={\sum }_{i=1}^{N}\min (\frac{\mathop{max}\limits_{j\in \{1..M\}\backslash m}({x}_{i},{\theta }_{j})\ast {\alpha }_{j}}{p({x}_{i}|{\theta }_{m})\ast {a}_{m}},1)$$

4 $$OverlapError=\mathop{{ma}{{x}}_{m=1...M}}\limits_{\,}(OverlapErro{r}_{m})$$

During the selection step, the individuals with high fitness values are chosen. The selection is not limited to just the best individuals but uses the tournament selection algorithm^9^ that allows a broader choice of individuals. A random number of individuals form a tournament to that one of them will win at random but weighted with the individual’s rank of fitness. Therefore, some less-fit individuals will be drawn into the next generation to enforce broader diversity of potential solutions. The selection will be repeated until the size of a generation is reached. The same individual can be selected repeatedly.

The mutation and recombination steps modify the individuals in each generation. During the mutation step, random individuals are selected and their parameters changed. Here, a random uniform mutation is employed. Therefore, a random number of parameters of an individual are chosen and then randomly drawn again within their respective minimum and maximum values. This process introduces the variety of solutions. The recombination step modifies individuals by choosing two random individuals and replacing them with their “children”. A pair of children ${c}_{1},{c}_{2}$ is defined by the weighted average of their parents ${p}_{1},{p}_{2}$ with a uniformly drawn weight $\alpha$ out of [0,1].

5 $${c}_{1}=\alpha {p}_{1}+(1-\alpha ){p}_{2}$$

6 $${c}_{2}=(1-\alpha ){p}_{1}+\alpha {p}_{2}$$

The recombination operation mainly fulfills the role of local optimization, which is why we have chosen the weighted average operation, while the mutation operation is used for introducing the variance.

Finally, the algorithm finishes after a fixed number of iterations by returning the GMM with the highest fitness.

### Implementation

Implementation of the “Distribution Optimization” evolutionary algorithm into the similarly named R library (https://cran.r-project.org/package=DistributionOptimization) makes use of the genetic algorithms provided by the R package “GA” (https://cran.r-project.org/package=GA^10^). The functionality can be accessed by the function of the same name: “DistributionOptimization()”. As input a data vector and the desired number of Gaussians are expected. As a balance between”OverlapError” and χ^2^ value is desired, a ratio between both in favor of the χ^2^ value may be set through the hyperparameter “OverlapTolerance”, which when set at its maximum value of 1 results in pure optimization of fitness. The general parameters defining the evolution can be set as provided by the “GA” package. Parameters for population size and the number of iterations may be chosen, depending on the difficulty of the task, and increased manually when necessary. The output consists of all parameters necessary to reproduce the final GMM, and of the seed needed to reproduce the evolutionary algorithm. The user can monitor the process through the “Monitor” parameter, which either silences the call or outputs stepwise fitness improvements. Further utilities needed for clustering, such as likelihood ratio test or the Akaike information criterion (AIC^11^), require the R Package “AdaptGauss” (https://CRAN.R-project.org/package=AdaptGauss^12^).

### Data sets and analyses

To assess the suitability of the “Distribution Optimization” for automated separation of the components of a GMM, the algorithm was applied on four different data sets available from published reports as specified below.

**Data set 1** is used as the main example data set to demonstrate the function of the algorithm and the usage of the associated R library. It comprises cold pain thresholds acquired from in n = 148 healthy volunteers^13^. Noxious cold stimuli had been applied using a 3 × 3 cm^2^ thermode placed on the volar forearm. The thermode was cooled down by −8 °C/s, starting from 32 °C. Following establishment of the individual cold pain threshold to stimulus with slower cooling (1 °C/s), where the subject had to indicate when the sensation changes from cool to painful, 11 stimuli with fast decreasing temperature were applied, ranging from −5 to +5 °C, in steps of 1 °C, from that threshold. Stimuli were applied at randomized order and the subjects rated each stimulus with respect to its painfulness. The “yes”/”no” responses were submitted to binary logistic regression to obtain the phasic cold pain threshold. The obtained pain thresholds to fast-cooling (“phasic”) thermal stimuli had been analyzed with respect to modal distribution. Specifically, the parameters of the GMM were optimized using the EM algorithm as implemented in our interactive R package “AdaptGauss”. The analysis had resulted in a trimodal distribution of phasic cold pain thresholds, with Gaussian modes located at mean temperatures of 24.5, 18.1 and 7.5 °C in decreasing order of cold pain sensitivity.

To determine the optimum number of components, model optimization had been done for *M* = 2 to 4 components, i.e., one mode less or more than in the original analysis. The final model was reestablished based on the Akaike information criterion. To test the robustness of the results, the “Distribution Optimization” algorithm was run 100 times using different values of “seed”. The 95% confidence intervals of the GMM parameter estimates were obtained as the 2.5^th^ and 97.5^th^ percentiles of the estimates from the 100 runs. This was compared to GMM fits in which the EM algorithm was used for model adaptation.

**Data set 2** consists of heat pain thresholds that had been used for the original publication of the interactive “AdaptGauss” R library^12^. The data had been obtained by locating a thermode at the skin of the forearm of n = 127 healthy volunteers and raising the temperature at 1 °C/s until the subject indicated a painful sensation. Interactive GMM analysis optimizing, based on the EM algorithm but with visually guided correction of the fit versus the observed PDE of the data, had identified a distribution pattern with M = 4 Gaussian modes located at temperatures of 32.3, 37.2, 41.4, and 45.4 °C.

**Data set 3** has been acquired in a cohort of n = 31 healthy subjects in whom the cortical excitability had been modulated by applying transcranial magnetic stimulation^14^. Following inhibitory stimulation of the primary motor cortex, the changes in the amplitudes of motor evoked potentials displayed a trimodal distribution with modes located at 69.7, 115.1 and 158.4%.

Finally, **data set 4** contains a sample of n = 10,000 microarrays derived gene expression values, observed in subjects with and without leukemia^15^. A total of 7,747 different Genes where assessed on 554 subjects, of whom 109 subjects where healthy while 445 patients were diagnosed with some kind of leukemia. Every sample represents the logarithmic intensity of a gene expression.

The automated “Distribution Optimization” GMM algorithm was run on these data sets and the obtained fits were compared with the original fits and with fits using the non-interactive standard EM algorithm implemented in the R library “mclust” (https://cran.r-project.org/package=mclust^16^). In addition, the quality of the fit was assessed by visual inspection of the fit and of a derived quantile-quantile (QQ) plot of the observed and predicted data distributions.

## Results

### Comparison of GMM fits using DistributionOptimization or the EM algorithm

**Figure 2:**
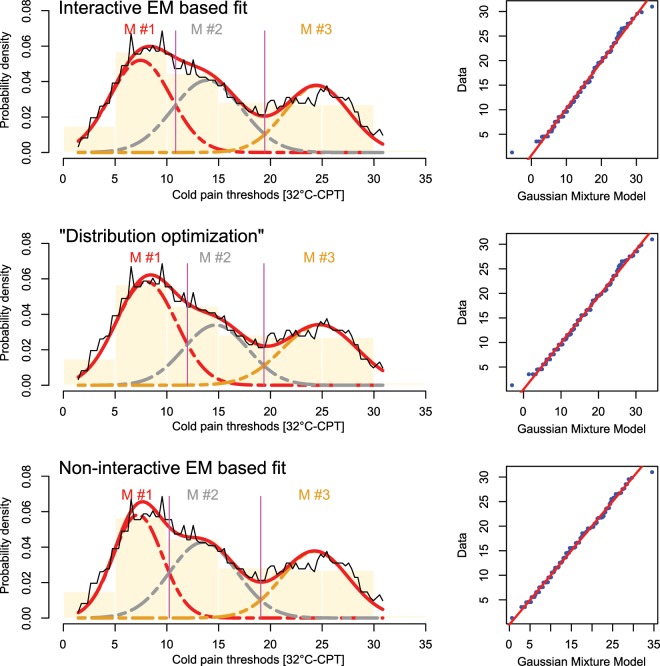
Fit of a GMM with M = 3 modes to **data set 1**(pain thresholds to cold stimuli acquired from healthy volunteers^13^). The distribution of the data is shown as probability density function (PDF) estimated by means of the Pareto density estimation (PDE^23^; black line) and overlaid on a histogram. The GMM fit is shown as a red line and the *M* = 3 single mixes are indicated as differently colored dashed lines (M#1, …, M#3). The Bayesian boundaries between the Gaussians are indicated as perpendicular magenta lines. At the right of the distributions, the respective QQ-plots are shown. Top: Original fit as published previously, obtained with an interactive EM based GMM adaptation^13^. Middle: Fit obtained with the automated “Distribution Optimization” algorithm. Bottom: Fit obtained using the EM algorithm without manual interaction. The figure has been created using the R software package (version 3.5.3 for Linux; http://CRAN.R-project.org/^24^) and the R libraries “AdaptGauss” (https://cran.r-project.org/package=AdaptGauss^12^) and “DistributionOptimization” (https://cran.r-project.org/package=DistributionOptimization).

The GMM fit of data set 1 using M = 3 modes as in the original publication^13^ resulted in mean values of m_1,…,4_ = 24.8, 14.7, and 8 °C. The “Distribution Optimization” algorithm well separated the three Gaussian modes, however, the Bayesian decision limits slightly differed from those obtained by either interactive or non-interactive GMM fit based on the EM algorithm (Fig. 2). In this data set, the interactive EM based fit had provided the best model solution according to an Akaike information criterion of AIC = 988.81. “Distribution Optimization” and non-interactive EM based fits were associated with values of the AIC of 994.96 and 992.36, respectively.

**Figure 3:**
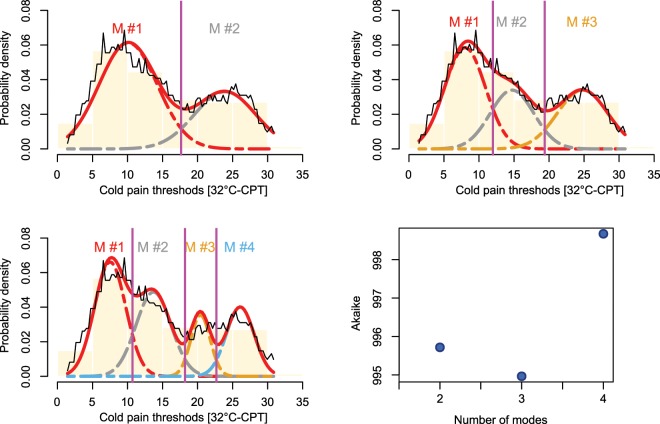
Assessment of a possible the number of modes in the distribution of **data set 1**. Fitting of a Gaussian (mixture) model (GMM) with M = 2, …, 4 modes (similar panels throughout the figure) to the distribution, shown as PDE (black line) and overlaid on a histogram. The GMM fit is shown as a red line and the M = 2, …, 4 single mixes are indicated as differently colored dashed lines (M#1, …, M#4). The Bayesian boundaries between the Gaussians are indicated as perpendicular magenta lines. The best fit was obtained with M = 3 modes (dots) as indicated by the lowest value of the Akaike in formation criterion. The figure has been created using the R software package (version 3.5.3 for Linux; http://CRAN.R-project.org/^24^) and the R libraries “AdaptGauss” (https://cran.r-project.org/package=AdaptGauss^12^) and “DistributionOptimization” (https://cran.r-project.org/package=DistributionOptimization).

**Figure 4:**
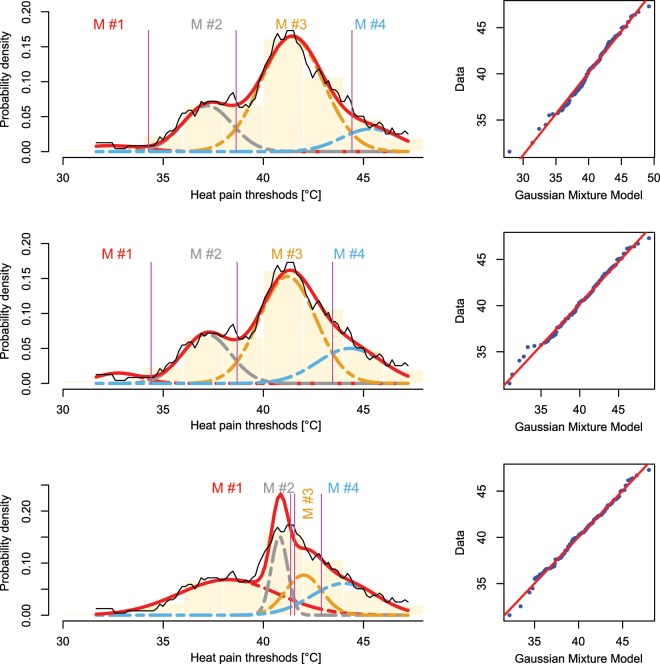
Fit of a GMM with M = 4 modes to **data set 2**. (pain thresholds to heat stimuli acquired in healthy volunteers^12^). The distribution of the data is shown as probability density function (PDF) estimated by means of the Pareto density estimation (PDE^23^; black line) and overlaid on a histogram. The GMM fit is shown as a red line and the M = 4 single mixes are indicated as differently colored dashed lines (M#1, …, M#4). The Bayesian boundaries between the Gaussians are indicated as perpendicular magenta lines. At the right of the distributions, the respective QQ-plots are shown. Top: Original fit as published previously, obtained with an interactive EM based GMM adaptation^12^. Middle: Fit obtained with the automated “Distribution Optimization” algorithm. Bottom: Fit obtained using the EM algorithm without manual interaction. The figure has been created using the R software package (version 3.5.3 for Linux; http://CRAN.R-project.org/^24^) and the R libraries “AdaptGauss” (https://cran.r-project.org/package=AdaptGauss^12^) and “DistributionOptimization” (https://cran.r-project.org/package=DistributionOptimization).

Fitting various GMMs to data set 1 showed that the “Distribution Optimization” algorithm always aims at least overlap among Gaussian modes (Fig. 3). The present experiment also indicted that reassessment of the originally trimodal distribution of cold pain thresholds verified the original M = 3 modes since this provided the lowest value of the AIC (Fig. 3 lower right panel). The focus of the “Distribution Optimization” algorithm on the separation of the Gaussian modes, disfavoring overlaps, became more evident in data set 2, where the non-interactive algorithm produced a solution with a counterintuitive separation of the subjects into clusters of the heat pain thresholds (Fig. 4). This is indicated by the location of the Bayesian decision limits that indicated a narrow second cluster not corresponding to the displayed data distribution. By contrast, the “Distribution Optimization” algorithm produced a GMM with means close to the original result obtained with the interactive EM based GMM fit, located at temperatures of 35, 37.2, 41 and 44.2 °C. Of note, while the solution provided by the non-interactive EM algorithm was unusable for topical interpretation, it provided nevertheless the lowest AIC criterion of AIC = 651.01, while the better grouping obtained with the interactive EM or “Distribution Optimization” based fits were associated with slightly higher values of AIC of 654.64 and 659.2, respectively.

**Figure 5:**
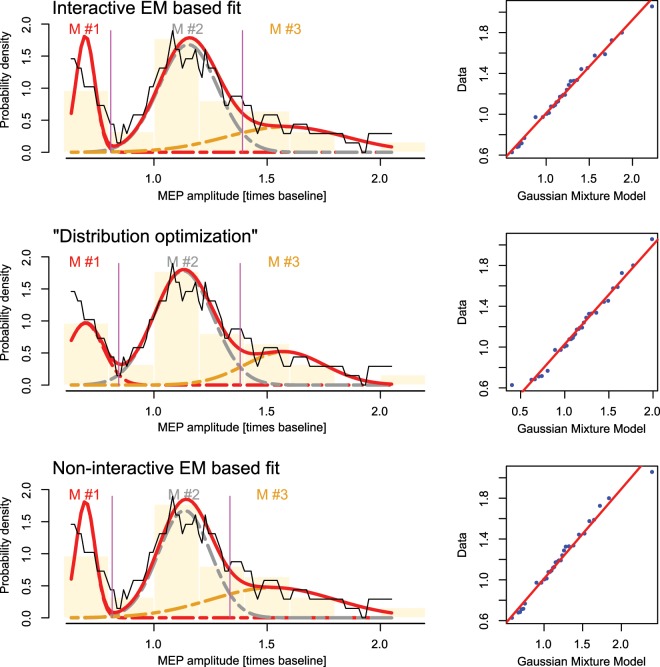
Fit of a GMM with M = 3 modes to **data set 3** (amplitudes of muscle potential evoked in healthy volunteers^14^). The distribution of the data is shown as probability density function (PDF) estimated by means of the Pareto density estimation (PDE^23^; black line) and overlaid on a histogram. The GMM fit is shown as a red line and the M = 3 single mixes are indicated as differently colored dashed lines (M#1, …, M#3). The Bayesian boundaries between the Gaussians are indicated as perpendicular magenta lines. At the right of the distributions, the respective QQ-plots are shown. Top: Original fit as published previously, obtained with an interactive EM based GMM adaptation^14^. Middle: Fit obtained with the automated “Distribution Optimization” algorithm. Bottom: Fit obtained using the EM algorithm without manual interaction. The figure has been created using the R software package (version 3.5.3 for Linux; http://CRAN.R-project.org/^24^) and the R libraries “AdaptGauss” (https://cran.r-project.org/package=AdaptGauss^12^) and “DistributionOptimization” (https://cran.r-project.org/package=DistributionOptimization).

**Figure 6:**
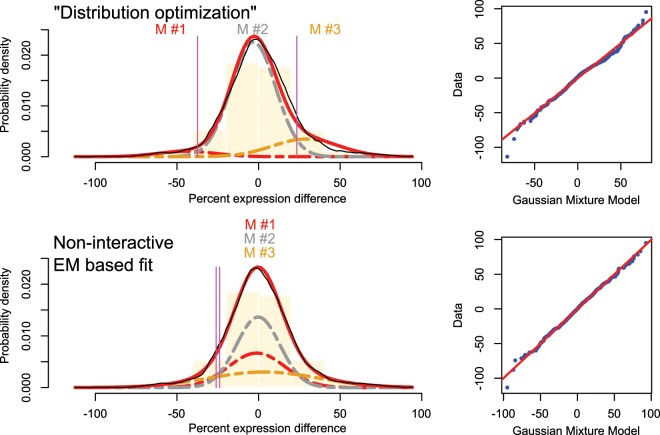
Fit of a GMM with M = 3 modes to **data set 4**. (microarray derived gene expression data form patients with leukemia and controls^15^). The distribution of the data is shown as probability density function (PDF) estimated by means of the Pareto density estimation (PDE^23^; black line) and overlaid on a histogram. The GMM fit is shown as a red line and the M = 3 single mixes are indicated as differently colored dashed lines (M#1, …, M#3). The Bayesian boundaries between the Gaussians are indicated as perpendicular magenta lines. At the right of the distributions, the respective QQ-plots are shown. Top: Fit obtained with the automated “Distribution Optimization” algorithm. Bottom: Fit obtained using the EM algorithm without manual interaction. The figure has been created using the R software package (version 3.5.3 for Linux; http://CRAN.R-project.org/^24^) and the R libraries “AdaptGauss” (https://cran.r-project.org/package=AdaptGauss^12^) and “DistributionOptimization” (https://cran.r-project.org/package=DistributionOptimization).

Fits of data set 3 again showed that the “Distribution Optimization” algorithm produced results comparable to those obtained with the alternatives tested in this analysis (Fig. 5). For data set 4, using M = 3 modes and aiming at their separation, “Distribution Optimization” terminated with Gaussian modes located at −44.1, −3.2 and 29% relative gene expression, whereas the raw EM based fit found almost superimposed modes with means at −1.1, −0.2 and 3% relative expression, and non-meaningful Bayesian decision limits (Fig. 6).

### Robustness of the distribution optimization results

When running 100 fits of data set 1 with M = 4 modes and setting the seed parameter at i in the i^th^ run, the “Distribution Optimization” algorithm produced parameter values of the GMM that varied between 3.3 and 12.9% (Table 1). By contrast, the EM algorithm produced the same results in every run, i.e., the between runs variance of the GMM parameter values was 0%.

**Table 1:** Parameters of the GMM obtained for data set 1, using 100 runs with different values of seed and either the “Distribution Optimization” or the EM algorithm (means, standard deviations, SD, and coefficients of variation, CV).

Parameter	Distribution Optimization			EM		
	Mean	SD	CV [%]	Mean	SD	CV [%]
µ_1_	8	0.3	3.3	7.2	0	0
µ_2_	15	0.9	5.9	13.5	0	0
µ_3_	24.9	0.5	2.1	24.3	0	0
σ_1_	2.8	0.3	9.6	2.4	0	0
σ_2_	3.2	0.4	11.4	3.3	0	0
σ_3_	3.6	0.4	11.3	3.2	0	0
α_1_	0.4	0	10.9	0.3	0	0
α_2_	0.3	0	11.1	0.3	0	0
α_3_	0.3	0	12.9	0.3	0	0

## Discussion

The proposed method of fitting GMMs with a focus on maximum separation of the single mixes while allowing least overlap, was successful in providing satisfactory fits of the probability density distributions of the data. The results were comparable with those obtained with classical EM based fits, except for data sets where the EM algorithm produced unsatisfactory results. The observation of such results as shown with data set 2 where the GMM provided no meaningful basis for group separation in a biomedical context had been the motive of the development of the present alternative GMM analysis method. Hence, differences to the automated EM based fit are expected. Of note, when using visually guided EM based GMM fitting, results were similar to those obtained with “Distribution Optimization”. Main advantages of the latter algorithm, however, are that it excludes the subjective component of visually guided fitting, is better suited for automated GMM fitting of many data sets, and makes the results better reproducible.

The present algorithm has been developed to replace algorithms that aim at maximizing the GMM’s likelihood, such as EM^2,3^, or an equivalent parameter of goodness-of-fit, such as used in Markov-chain Monte-Carlo algorithms^4^. EM is divided into an expectation and a maximization step, which are repeated to incrementally improve the likelihood of the model. The algorithm stops when the improvement falls under a certain threshold. During the expectation step, the priori likelihood is calculated based on the current model parameters. A likelihood function can therefore be constructed, based on a classification of every data point to one of the GMM components with the highest Bayesian probability. The maximization step then maximizes the parameters, given this information. For the general problem of local maxima, many different modifications of the algorithm have been proposed. For example in CEM^2,17^ and ECM^18^, only a single component is maximized in each iteration. SMEM^19^, SSMEM^19^ and Competitive EM^20^ are variants of EM that include split and merge operations on GMM components. Randomly mutating EM algorithms are the Random Swap EM^21^ or the genetic based EM. However, these algorithms do not aim at replacing the EM algorithm but rather at reducing one of its weaknesses, the risk of ending in local and not global optima. An alternative to EM are the Markov-chain Monte-Carlo algorithms^4^. They nevertheless follow the same idea as EM by alternately determining weightings, using Bayesian classification, and deriving expectation values and standard deviations from those weightings. Instead of maximizing those values in each iteration, Monte Carlo sampling is used, and the final GMM is given by the stationary distribution of the Markov chain. This, however, equals in a maximization of likelihood as in the EM algorithm. All covered methods ultimately only differ in in their way of searching the maximal likelihood, which we argue is not necessarily the best or most appropriate measure.

The reproducibility of the results, however, was lower than that obtained when using classical EM based fitting, as shown in the robustness experiment. This owes to the genetic algorithm being naturally dependent on a degree of randomness. This however allows an overcoming of local maxima and has the advantage of approximating different solutions. This allows for an automated generation of multiple significant models, which can offer different interpretations and has the effect that the fits differ from those obtained with EM. Specifically, the effect demonstrated in the sample data sets is mainly caused by the choice of quality measures during the fitting process. We have shown that statistically sound GMM can be reached by optimization of a different quality measure than likelihood and that an additional quality can be introduced, producing a model that is significantly different for classification purposes than what is reached by EM. The problem search space for complex problems as GMM is not transparent and there is always explicit or implicit bias introduced through such optimizations, one can never ultimately conclude that there are no models that reach higher performance under the quality measures with a less desirable structure.

The preference of “Distribution Optimization” to EM based alternative methods for GMM fitting not only depends on the reproducibility of the results, the goodness of fit, and the robustness of the results, but has a clear contextual component. This is shown with data set 4. The EM fit of a GMM consisted in a mixture of almost superimposed Gaussian modes with nearly identical means but different standard deviations (Fig. 6). By contrast, “Distribution Optimization” produced three distinct modes with a main mode in the center and two modes of low weights at its margins. In biomedical research such represented in the leukemia derived data set 4, the usual criterion to define an effect is a difference in mean, for example as defined in the effect size measure of Cohen’s d^22^. Research aims often at the identification of subgroups in the data, and these subgroups are characterized by different central values of the selection parameter, such as different means. With the EM based fit, a useful group separation was impossible whereas the “Distribution Optimization” provided this readily. However, when group separation is not a topical focus and by contrast, the data are considered to represent groups with similar means but different variances, “Distribution Optimization” would be unsuitable while EM based GMM fitting may provide the desired result. Thus, the choice of the method should consider both, the statistical soundness and the topical context.

By its optimization toward mode separation, the proposed “Distribution Optimization” evolutionary algorithm for GMM fitting provides a suitable basis for group separation in multimodally distributed data. It introduces choice between multiple significant models, which would not have occurred by pure means of likelihood optimization such as in alternative approaches to GMM fitting. When group separation is intended, the “Distribution Optimization” algorithm may outperform alternative EM based methods in some data sets.

## Data Availability

The “DistributionOptimization” R package is freely available at https://cran.r-project.org/package=DistributionOptimization.

## References

[1] Ameijeiras-Alonso, J., Crujeiras, R. M. & Rodríguez-Casal, A. Mode testing, critical bandwidth and excess mass. *ArXiv e-prints* (2016).

[2] Dempster AP, Laird NM, Rubin DB (1977). Maximum Likelihood from Incomplete Data via the EM Algorithm. *Journal of the Royal Statistical Society*. Series B.

[3] Bishop, C. *Pattern recognition and machine learning*. (Springer, 2006).

[4] Frühwirth-Schnatter, S. *Finite Mixture and Markov Switching Models*. (Springer New York, 2006).

[5] Kim DK, Jeremy MGT (1995). The Restricted EM Algorithm for Maximum Likelihood Estimation Under Linear Restrictions on the Parameters. Journal of the American Statistical Association.

[6] Bayes M, Price M (1763). An Essay towards Solving a Problem in the Doctrine of Chances. By the Late Rev. Mr. Bayes, F. R. S. Communicated by Mr. Price, in a Letter to John Canton, A. M. F. R. S. Philosophical Transactions.

[7] Keating JP, Scott DW (1999). A Primer on Density Estimation for the Great Home Run Race of 98. Stats.

[8] Ultsch, A. Optimal density estimation in data containing clusters of unknown structure. Technical Report No. 34. (Dept. of Mathematics and Computer Science, University of Marburg, Marburg, Germany, 2003).

[9] Goldberg, D. & Deb, K. A comparative analysis of selection schemes used in genetic algorithms. *Foundations of Genetic Algorithms* (1991).

[10] Scrucca L (2013). GA: A Package for Genetic Algorithms in R. Journal of Statistical Software.

[11] Akaike H (1974). A new look at the statistical model identification. IEEE Trans. Aut. Control.

[12] Ultsch A, Thrun MC, Hansen-Goos O, Lötsch J (2015). Identification of Molecular Fingerprints in Human Heat Pain Thresholds by Use of an Interactive Mixture Model R Toolbox (AdaptGauss). Int. J. Mol. Sci..

[13] Weyer-Menkhoff I, Thrun MC, Lotsch J (2018). Machine-learned analysis of quantitative sensory testing responses to noxious cold stimulation in healthy subjects. Eur. J. Pain.

[14] Heidegger T, Hansen-Goos O, Batlaeva O, Ziemann U, Lötsch J (2017). A data-driven approach to responder subgroup identification after paired continuous theta burst stimulation. Front Human Neurosci.

[15] Thrun, M. C. & Ultsch, A. Quality Measurements of Projections to Evaluate Discontinuous Structures of High-dimensional Data. *Journal of Machine Learning Research* **17** (2016).

[16] Scrucca L, Fop M, Murphy TB, Raftery A (2016). E. mclust 5: clustering, classification and density estimation using Gaussian finite mixture models. The R. Journal.

[17] Celeux G, Chretien S, Forbes F, Mkhadri A (2001). A Component-Wise EM Algorithm for Mixtures. Journal of Computational and Graphical Statistics.

[18] Meng X-L, Rubin DB (1993). Maximum likelihood estimation via the ECM algorithm: A general framework. Biometrika.

[19] Wang HX, Luo B, Zhang QB, Wei S (2004). Estimation for the number of components in a mixture model using stepwise split-and-merge EM algorithm. Pattern Recognition Letters.

[20] Zhang B, Zhang C, Yi X (2004). Competitive EM algorithm for finite mixture models. Pattern Recognition.

[21] Zhao Q, Hautamki V, Krkkinen I, Frnti P (2012). Random swap EM algorithm for Gaussian mixture models. Pattern Recognition Letters.

[22] Cohen, J. A coefficient of agreement for nominal scales. *Educ. Psychol. Meas*. **20** (1960).

[23] Ultsch, A. In *Innovations in Classification, Data Science, and Information Systems - Proceedings 27th Annual Conference of the German Classification Society* (GfKL). (eds. Baier, D. & Werrnecke, K. D.) (Springer).

[24] R Development Core Team. R: A Language and Environment for Statistical Computing. (2008).

